# Farmer’s traditional practices in milk and milk products handling in selected districts of North Wollo, Ethiopia

**DOI:** 10.1016/j.heliyon.2024.e40866

**Published:** 2024-12-03

**Authors:** Areaya Gelaw, Solomon Abegaz, Nurlgn Mohammed, Mengie Ahmed

**Affiliations:** a Department of Animal Sciences, College of Agriculture, Woldia University, Mersa, Ethiopia; b Department of Animal Science, College of Veterinary Medicine and Animal Science, University of Gondar, Gondar, Ethiopia

**Keywords:** Dairy, peri-Urban, Production system, Rural, And urban

## Abstract

Dairy production is practiced almost all over Ethiopia. Milk and milk products play an important role in human nutrition throughout the country but the quality of milk produced in Ethiopia is poor and below the standard due to poor pre-milking and postharvest handling practices. The study was carried out from October 2022 to May 2023 with the objective of assessing and document the traditional practices of farmer's milk and milk products handling in selected districts of north Wollo, Ethiopia. A cross-sectional study was undertaken on 254 using stratified random sampling technique. Data were collected using semi structured questionnaire, observation, focused group discussion. Frequency of milking in morning and evening, morning only and evening only were significantly different (P < 0.001) in three dairy production system. Majority of respondents milked their cows twice a day (morning and evening milking) 68 %, 70.8 %, and 46.4 % in urban, peri-urban and rural dairy production system, respectively. The remaining 32 %, 29.2 %, and 53.6 % respondents in urban, peri-urban and rural production system were milked their cows once a day either morning only or evening only, respectively. The majority of smallholders in urban and peri-urban dairy production systems use plastic vessels for milk and milk products handling, whereas, rural smallholders used bottle gourds. According to respondents, materials use for cleaning like plants, detergents and, water alone were significantly different (P < 0.001) in the dairy production system. The purpose of smoking milk utensils was believed to increase the shelf life of dairy products 32 % 24.7 %, and 46.4 % in urban, peri-urban and rural production system, respectively. Therefore, supporting of farmer's traditional practices via training, identification of plants that are used for smoking and introduction of milk cans for farmers are crucial for improvement of milking and milk handling practices.

## 1 Introduction

Ethiopia stands first in Africa and fifth in the world with cattle population endowment which is estimated to be about more than 66 million heads of cattle. Out of total cattle population, cows represent about 56.69 % and the remaining 44 % are male animals; as well as about 96.93 % of the total cattle in the country are local breeds [1]. Crossbreed cows produce more milk than local cows due to their genetic potential ([2], [3]) however, out of the total dairy cattle keepers in the country 96.93 %, keep on local breeds types which are poor genetic potential for milk production [1]. The remaining 2.67 % and 0.40 % are crossbreed and exotic cattle breeds respectively [1]. According to Ref. [1] average lactation period per cow is 7 months and milk production averages 1.48 L per day/cow.

Dairy plays important role in different agricultural activities and socio-economic aspects of people, such as milk for human consumption and as a source of cash income [4]. Dairy production is practiced almost all over Ethiopia involving a vast number of small subsistence farms and is one of the major economic development contributors ([5]; [6]).

Ethiopia has a shortage of dairy products and imports dairy products from other countries ([7]). The low production of milk in the nation calls for improving the productivity of dairy cattle, animal health (udder and reproductive health). The quality of milk produced in Ethiopia is poor and below the standard due to poor pre-milking and post-harvest handling practices and highly characteristics of the milk perishability (Tsadikan and Gurja, 2018; [8]).

Despite its potential for growth and economic benefits, Ethiopia's dairy industry is underdeveloped and traditional in the majority of the country [9]; [3,10]. In the study area there is increasing demands for dairy products from time to time due to increasing human population, urbanization and increase in household income (North Wollo Livestock and Fishery Resource Office).

Previous research conducted in Ethiopia indicated that the poor quality of milk is attributed to inadequate pre- and post-harvest milk handling practices and the highly perishable nature of milk ([11]). Improper handling and disregard of hygienic measures by milk handling personnel may favor undesirable microbes to contaminate milk survive and multiply and make milk unsafe for both direct consumption and further processing ([12]).

Smallholder farmers handle milk and milk products according to their own customs. In the study areas, however, there is a lack of documentation regarding the indigenous knowledge of farmers. Therefore, the objective of this study was to identify and document the traditional practices of farmer's milk and milk products handling in selected districts of north Wollo, Ethiopia.

## 2 Materials and methods

### 2.1 Description of study areas

**Fig. 1:**
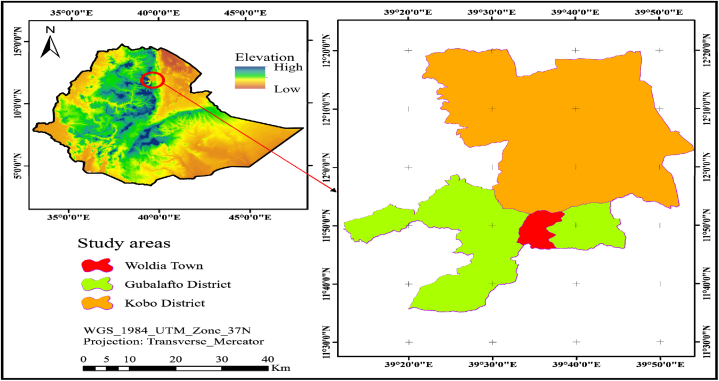
Map of study areas.

The research was carried out in a few districts north of Wollo. The map of the study area showed in (Fig. 1). The study areas were classified into three main production system; urban, peri-urban, and rural productions based on their location in Ethiopia. The definition of production system was:

1. Urban dairy production system is a purely market-oriented production system located within cities. Urban milk production system is settled in major cities, which have a high demand for milk and they are a largest source of milk producer [13].
2. Peri-urban dairy production system is a purely market-oriented production system but located around or close to the boundaries of cities. Urban and per-urban milk production system involves the production, processing and marketing of milk and milk products into urban centers [13].
3. Rural dairy production system is part of the subsistence farming system that contributes up to 98 % of the total milk production of in Ethiopia, and not market oriented [14]; [15].

The study area is expressed by high local zebu cattle with population of 977,376 cattle, from which 433,422 are male and 543,954 are females. The representative districts of north Wollo were Woldia town, Gubalafto and Raya Kobo district. Woldia town, Gubalafto and Raya Kobo district represent urban, peri-urban and rural dairy production systems, respectively. According to Ref. [1] report north Wollo zone has a total of 17.8 thousand dairy heads of cows and 174.7 thousand heads of milking cows, the average lactation period is five months and milk production averages 1.39 L per day/cow. The study area is characterized by high human population with increasing demand for dairy products, large livestock resources and dairy products are used as one of the means of livelihood strategies of the community.

Woldia town is located at about 11° 50′ N latitude and 39° 46′ E longitude with an elevation 2112 m above sea level, 22°c average temperature and the mean annual rainfall ranging from 650 to 850 mm. Gubalafto district is located at about 12° 00′ N latitude and 39°19′ E longitude with monthly temperature ranges between 21°c and 25°c.

Raya Kobo district is located at about latitude of 12°09′ and 39°38′ E of longitude with an elevation of 1468 m above sea level. Ray Kobo obtains minimum and maximum annual rainfall of 500 and 800 mm, respectively. The area has minimum and maximum annual temperature of 12 and *33*^*0*^*C* according to Raya Kobo district Livestock and Fishery Resource Office unpublished data.

### 2.2 Study population and sampling techniques

The survey was conducted using a multi-stage sampling technique. Based on the potential for dairy production and the experiences of smallholder farmers, three study areas Woldia, Gubalafto, and Raya Kobo were specifically chosen from north Wollo for the first stage. In the second stage, three sub cities were selected from Woldia town, five kebeles from Gubalafto and six kebeles from Raya Kobo districts were purposely selected based on dairy cattle population. Altogether, 576 male and 120 female (696 households) were identified according to formula described Yemane (1967). Having dairy cows were identified based on North Wollo Zone livestock and Fishery Resource Development Office report. In the third stage, then, 254 study respondents having at least one local or cross breed milking cows were purposely selected from 696 households. Number of respondents in the study areas were presented in (Table 1). The formula used to determine the sample size was showed in equation (1)

(1) $$n=\frac{N}{1+e^{2}N}$$

Where,

**Table 1:** Number of respondents in the study areas.

S/N	Production System	Total Population			Sample Size of the survey
		Male	Female	Total	
1	Urban	49	20	69	25
2	Peri-urban	206	37	243	89
3	Rural	321	63	384	140
	Total	576	120	696	254

n = sample size.

N = population size

e = sampling error (0.05)

2 = raised the power of.

Sample size (n) $=\frac{696}{1+{e0.05}^{2}696}$ = 254 study respondents. The total samples of households included in the study were determined by the formula given by Yamane [16] with 95 % confidence level.

### 2.3 Methods of data collection

Primary data was obtained from dairy producers using semi-structured pre-tested questionnaire and focused group discussion. Information was obtained on dairy products handling and hygienic practices. And primary data were supported by secondary data sources. The questionnaire was administered by recruited enumerators (diploma holders in Animal Science who were trained before actual data collection).

Focused group discussions were conducted in urban, peri-urban, and rural production systems in the study areas to clarify issues that were not well addressed during the questionnaire interview and to validate information provided by individual study respondents. Eight individuals consisting of men and women households have participated in the focused group discussions.

Field observation was carried out to validate information provided from primary and secondary sources.

### 2.4 Data analysis

All data collected from study areas were analyzed using Statistical Package for Social Sciences ([17]) software, version 20. Descriptive statistics such as percentage, mean and standard deviation, chi square, and X^2^ test were used to summarize the results. Statistical significance between variables was examined using P-values at critical probability of P < 0.05.

## 3 Results and discussion

### 3.1 Milking and milk handling practices

The results of milk handling practices are showed in (Table 2). Frequency of milking in morning and evening, morning only and evening only were significantly different (P < 0.001) in three dairy production system. In the study areas, hand milking was the sole milking method and calves were allowed to suckle their dams before and after milking in all of the study areas. However, in urban areas (23 %) of the dairy producers bucket feeding for calves. According to the respondents, in urban production systems 68 %, 24 % and 8 % of respondents milked their cows in the mornings and evenings, evenings only, and mornings only, respectively. In rural production systems 46.4 %, 35 % and 18.6 % of respondents reported that they milked their cow during mornings and evenings, evenings only and mornings only, respectively. In the peri-urban dairy production systems, 70.8 %, 19.1 % and 10.1 % of the study respondents that they milked their cows during morning and evening, evening only, and morning only respectively. All (100 %) of the study respondents cleaned their hands and milking utensils before milking.

**Table 2:** Frequency of milking and milking procedure in the study areas.

Variables	Urban		Peri-urban		Rural		Chi square	P-value
	N	%	N	%	N	%		
**Frequency of milking**								
Morning and evening	17	68	63	70.8	65	46.4		
Morning only	6	24	17	19.1	49	35		
Evening only	2	8	9	10.1	26	18.6	35.12	<0.001∗∗
**Procedure of milking**								
Washing of hands and vessels only	–		–		140	100		
Washing of udder and teats before milking	16	64	72	80.9	–			
Do not wash udder and teats	9	36	17	19.1	–	–	28.74	<0.001∗∗
**Use of towels**								
Use of collective towels	10	40	45	50.6	–			
Use of individual towels	6	24	27	30.3	–			
Do not use towels	9	36	17	19.1	140	100	64.25	<0.001∗∗

Procedures of milking like washing of hands and vessels only, washing of udder and teats before milking, and do not wash udder and teat were significantly different (P < 0.001) in urban, peri-urban and rural dairy production system. According to respondents, about 64 % of the respondents were wash udder and teats of cows before milking in urban production system. The remaining 36 % of respondents were do not wash udder and teats. About 40 % and 24 % of the study respondents used collective and individual towels for washing udder and teats of cow, respectively. But 36 % of the study respondents indicated that they do not use towels at all. During milking time, 80.9 % washed udder before milking, 19.1 % do not wash udder and teats of cows. Based on the respondents, use of collective towels, use of individual towels, and do not use towels were significantly different (P < 0.001) in the dairy production system. About 50.6 % and 30.1 % of the study respondents used collective and individual towels respectively, but 19.1 % of respondents reported that they do not use towels at all in the per-urban dairy production system. This study is in agreement with [18]; [19] that revealed that hygienic milk handling practices such as washing udder with potable water, cleaning milking barn, drying of udder and teats using with individual towels, cleaning and sanitizing of hands and milking utensils and milking storage containers use of dirty water for cleaning of udder and teats absence of cold chain, use of in appropriate transportation vehicles and unhygienic retailing practices predisposes milk to microbial contamination.

Milking practice in the current study is in agree with the reports of Tadesse et al. [20] that revealed that 96.8 % of farmers in Abuna Gindeberet districts milked their cows twice a day and 3.2 % of them milked their cows once a day during dry seasons when feed is scarce. Ayalew [21] also indicated that cows were milked twice a day in urban, peri-urban and rural areas during early and mid-lactation season in south Wollo zone.

Cleaning of udder and teats before milking contributes to hygienic milk production. However, it is not common practice to sanitize teats before milking in the rural production systems, and the number of farmers who clean udder and teats of cows is few in urban and peri-urban dairy production systems. According to the respondents, there is an assumption that udder and teats of milking cows are cleaned when calves are allowed to suckle before milking. However, this might be a potential source for cross contamination of milk with microorganisms from the oral cavity of calves.

### 3.2 Milk vessels used for milking and processing of dairy products

The result of utensils used for milking, storage and churning of dairy products in the study areas are presented in (Table 3). According to study respondents in the current finding all respondents (100 %) in urban and peri-urban areas used plastic containers whereas, majority of (74.3 %) study respondents in rural areas used bottle gourds for milking purposes. Contradict from this finding about 45 % of rural producers used clay pots, 45 % used plastic utensils and the remaining 10 % used aluminium utensils for milk handling Asratet et al., 2015. In urban (32 %), peri-urban (39.3 %) and rural areas (81 %) clay pots are used for churning purposes. The respondents indicated that in urban and peri-urban areas milk is churned only during main fasting season of (*Abiy Tsom or hudade)* of the year by members of the Ethiopian Orthodox religion during which the demand for raw milk is low.

**Table 3:** Milk vessels used for various purposes in the study areas.

Production system	Types of materials	% of Respondents				
		Bottle gourd	Clay pots	Bottle gourd & Clay pots	Plastic containers	Stainless steel
Urban	Milking	–	–	–	100	–
	Churning	12	32	13	43	–
	Storage	7.3	–	–	80	12.7
Peri-urban	Milking	–	–	–	100	–
	Churning	23.8	39.3	–	25.8	11.1
	Storage	13.5	–	–	60.7	25.8
Rural	Milking	74.3	–	–	25.7	–
	Churning	81	4.7	14.3	–	–
	Storage	55.7	35	–	9.3	–

In urban (80 %) and peri-urban (60.7 %) areas plastic materials are used for storage of milk and 55.7 % of respondents in rural areas used bottle gourds used for the same purpose.

### 3.3 Materials used for cleaning and smoking of milk utensils

The results of plant materials used for cleaning and smoking of milk utensils in the study areas are presented in (Table 4). According to respondents, materials use for cleaning like plants, detergents and, water alone were significantly different (P < 0.001) in the dairy production system. Almost all of the smallholder dairy farmers (92 %, 91 %) in urban and peri-urban and majority (67.15 %) in rural areas reported that they wash milking utensils before and after every use. All of the respondents in urban and 58.4 % of those in peri-urban areas washed their milk containers using water and detergents, whereas in rural areas (80 %) of respondents used different plants such as “*kessie*, *Chifrig*, *Kibie zilzil*, and *Beles kitel*” for washing of milking and storage utensils. Similar findings were found in rural areas, where the majority of respondents (93.3 %) typically use various plant species to clean their milking utensils before and after milking. The remaining respondents clean their utensils once a day after milking because they keep them dry and clean after use, according to Asratet et al. [22]. This study is in line with Alemu and Girma (2018) that revealed that plants used for cleaning and washing of milk utensils were *Kessie (Lippia adoensis)*, *Weynagift (Senecio myriocephalus)* and *Chifirig (Sida cuneifolia)* around Dessie administrative region of south Wollo zone of northern Ethiopia.

**Table 4:** Cleaning and smoking vessels in the study areas.

Variables	Urban (N = 25)		Peri-urban (N = 89)		Rural (N = 140)		Chi square	P-value
	N	%	N	%	N	%		
**Washing of milk containers**								
Before use	–		3	3.4	33	23.6		
After use	2	8	5	5.6	13	9.3		
Both	23	92	81	91	94	67.1	93.91	<0.001∗∗
**Materials used for cleaning purposes**								
Plants	–		37	41.6	112	80		
Detergents	25	100	52	58.4	21	15		
Water alone	–		–		7	5	76.27	<0.001∗∗
**Plants for smoking**								
Woira	25	100	60	67.4	106	75.4		
Kitkita	–		29	32.6	34	13.4	78.37	<0.001∗∗
**Purpose of smoking**								
Believed to prolongs shelf life of dairy product	8	32	22	24.7	65	46.4		
Imparts good aroma	15	60	62	69.7	52	37		
Imparts orange color to butter to be made from fermented milk	2	8	5	5.6	23	16.4	102.28	<0.001∗∗

N = Number of respondents.

All (100 %) of respondents indicated that they smoke milking and storage utensils for different reasons. The major reasons indicated by the study respondents were for importation of good flavor (50.8 %), longer shelf life (37.4 %) and to get orange colored and attractive odor of butter (11.8 %). All of the respondents in urban (100 %), 67.4 % of them in peri urban and 75.4 % in rural dairy production setting reported that they use ‘*Woira’* (*Olea Africana*) and the rest 32.6 % and13.4 % of respondents in peri urban and rural areas revealed that they use ‘*kitkta’* (*Dodonaea angustifolia*) for smoking of milk vessels. The current finding is in line with Alemu and Girma [23], in south Wollo zone, Tsadikan and Gurja (2018) in Northern Ethiopia and Teshome and Tesfaye (2017) in Bench Maji Zone.

### 3.4 Patterns of consumption and utilization of dairy products in the study areas

The results of patterns of consumption and utilization of dairy products in the study areas depicted in (Table 5). In 8 %, 31.5 %, and 72.9 % of cases, buttermilk or fermented milk is used for human consumption. Dairy production systems in urban areas use fresh milk 36 % of the time, peri-urban areas use 3.4 %, and rural areas use 12.9 %. Fermented milk is used 40 % of the time, 9 % of the time, and 10.7 % of the time. Butter is used for consumption by 28 %, 12.4 %, and 14.2 % of study participants, respectively, and for income generation (64 %) according to urban respondents, 70.9 % of peri-urban respondents, and 57.9 % of rural respondents. The remaining percentages of study participants 8.8 %, 16.8 %, and 27.9 %, respectively, use butter for ointment for hair, particularly among study participants who are female. This finding is similar with Asratet et al. 2015, who reported in rural areas, butter was the primary product for 80 % of the households and cottage cheese for the remaining 20 % of the households. This finding is also in agreement with Ayalew [21] who reported that fresh milk was consumed (6.7 %, 14.4 %) and fermented milk was consumed (11.7 %, 18.9 %) in rural and urban dairy production respectively.

**Table 5:** Types of dairy products and their utilization patterns in the study areas.

Type of dairy products and their use	Urban (N = 25)		Peri urban (N = 89)		Rural (N = 140)		Overall (N = 254)	
	N	%	N	%	N	%	N	%
Fresh milk	9	36	3	3.4	9	12.9	21	8.3
Fermented milk	10	40	8	9	15	10.7	33	13
Buttermilk	2	8	28	31.5	89	72.9	119	46.9
All of the above	4	16	50	56.2	27	16.4	81	31.9
**Uses of butter**								
Income generation	16	64	63	70.9	81	57.9	160	63
Ointment for hair	2	8	15	16.8	39	27.9	56	22
Consumption	7	28	11	12.4	20	14.2	38	15

N= Number of respondents.

### 3.5 Marketing of milk and milk products in the dairy production systems

The results of marketing of milk and milk products in the dairy production systems are shown in (Table 6). According to the study respondents, fresh milk (60.0 ±0 .00) and butter (465.75 ± 36.476) are the most marketable products through the informal marketing system. Demand for dairy products in the study areas depends on fasting and non-fasting periods of the Ethiopian Orthodox religion believers. Milk producers, milk cooperatives, and consumers were key participants in the milk market. Cooperative marketing channel is exceptional to peri-urban system. Similarly, milk producers, retailer traders and consumers were key participants in the butter market.

**Table 6:** The average selling price (ETB) of milk (litters) and butter (in Kg) in the study areas.

Selling price	Urban (N = 25)	Peri-urban (N = 89)	Rural (N = 140)	Overall Mean ± SD	p-value
	Mean ± SD	Mean ± SD	Mean ± SD		
Fresh Milk	60.00 ±0 .00	60.0 ±0 .00	–	60.0 ±0 .00	–
Butter	504.00 ±0 .30.0^a^	485.39 ± 19.543^b^	446.43 ± 33.738^c^	465.75 ± 36.476	0.00

N=Number of respondents; SD=Standard Deviation.

During the survey period, average price of milk was 60.00 ETB/liter in urban and peri-urban areas, whereas in rural areas milk was not sold. The average price of butter was 504.00 ± 30.00, 485.39 ± 19.543 and 446.43 ± 33.738 ETB/Kg in urban, peri-urban and rural areas, respectively.

The selling price of butter in urban areas was significantly higher (p < 0.05) than in peri-urban and rural dairy production systems. It suggests that butter consumption is higher in urban areas than in dairy production systems.

## 4 Conclusions

Hand milking is the primary method of milking, according to the study's findings. Calves are permitted to nurse their dams both prior to and following milking. Before milking, all farmers wash their hands and utensils. Before milking, the majority of farmers in urban and peri-urban areas wash their udders and teats, but not in rural areas. Bottle gourds are crucial for rural areas, but plastic containers are the main milking material in urban and peri-urban areas. The most crucial supplies for handling milk and milk products are food-grade milk cans, which farmers cannot afford. To ensure that their butters had a long shelf life, a pleasant odor, and an orange hue, all farmers used locally grown plants to store their utensils both before and after milking. In terms of milk and milk handling techniques, urban and peri-urban farmers' indigenous knowledge is comparable to that of rural farmers, but slightly different. Therefore, improving milking and milk handling practices requires introducing milk cans for farmers, identifying plants used for smoking, and supporting their indigenous knowledge through training [8].

## CRediT authorship contribution statement

**Areaya Gelaw:** Software, Project administration, Methodology, Investigation, Formal analysis, Data curation, Conceptualization. **Solomon Abegaz:** Writing – review & editing, Writing – original draft, Visualization, Validation, Supervision, Conceptualization. **Nurlgn Mohammed:** Validation, Supervision, Software, Project administration, Methodology, Investigation, Formal analysis, Data curation, Conceptualization. **Mengie Ahmed:** Writing – review & editing, Writing – original draft, Validation, Supervision, Software, Conceptualization.

## Data availability statement

Data available up on reasonable request from corresponding author.

## Declaration of competing interest

The authors declare that they have no known competing financial interests or personal relationships that could have appeared to influence the work reported in this paper.
